# Supplementary material for: Content-rich biological network constructed by mining PubMed abstracts
Source: BMC Bioinformatics. 2004 Oct 8;5:147. doi: 10.1186/1471-2105-5-147 (PMC528731; doi:10.1186/1471-2105-5-147)
Supplement: Additional File 2 — The original results of the above study (non-essential files are deleted to keep the file size under the limit set by BMC bioinformatics). [file 1471-2105-5-147-S2.bz2 › chilibotAdditionalFile2/dip05/489ID10400640E10649/html/AR_RAN.html]

 


 **AR** and **RAN** 
  
Found 12 abstracts in PubMed, retrieved 05.  
 

 What does Google say? 
 PDF only 
| .edu only 

---

**Interactive relationship** (e.g. stimulation, inhibition, etc)

**Neutral relationship**- Here we have identified  **Ran**  binding protein in the microtubule organizing center RanBPM to be a novel  **AR**  interacting protein by yeast two hybrid assay and have confirmed this interaction by glutathione S transferase and His tagged pull down assays.  Ref: 12361945 J Biol Chem, 2002

**Non-interactive relationship** (e.g. studied together, co-existance, homology, etc.)

- Our results revealed nearly constant expression of  **AR**  and heterogeneous expression of  **AR**  co factors, with increased expression of PIAS1 and  **Ran**  ARA24  [ **RAN** ] , decreased expression of ELE1 ARA70, and no change in TMF1 ARA160, ARA54, SRC1, or TRAP220.  Ref: 12368219 Am J Pathol, 2002
- ARA267 alpha can also enhance  **AR**  transactivation with other coregulators, such as ARA24  [ **RAN** ]  or PCAF, a histone acetylase, in an additive manner.  Ref: 11509567 J Biol Chem, 2001
- We demonstrate by DNA microarray, RT PCR, Western blot and immunofluorescence analyses that treatment of androgen sensitive prostate cancer cells LNCaP with 10 5 M RE for 48 hr downregulates prostate specific antigen PSA,  **AR**  co activator ARA 24  [ **RAN** ]  and NF kB p65.  Ref: 12569576 Int J Cancer, 2003
